# Bone mineral density and its influencing factors in Chinese children with spinal muscular atrophy types 2 and 3

**DOI:** 10.1186/s12891-021-04613-x

**Published:** 2021-08-24

**Authors:** Xiaoyin Peng, Yujin Qu, Xiaohui Li, Junting Liu, Xinying Shan, Jia Wang, Fang Song

**Affiliations:** 1 Department of Neurology, Children’s Hospital Capital Institute of Pediatrics, Beijing, 100020 People’s Republic of China; 2 Graduate School of Peking Union Medical College, Beijing, 100730 People’s Republic of China; 3 Department of Medical Genetics, Capital Institute of Pediatrics, No 2, Ya Bao Road, Beijing, 100020 People’s Republic of China; 4 Department of Cardiology, Children’s Hospital Capital Institute of Pediatrics, No 2, Ya Bao Road, Beijing, 100020 People’s Republic of China; 5 Department of Epidemiology, Capital Institute of Pediatrics, Beijing, 100020 People’s Republic of China

**Keywords:** Spinal muscular atrophy, Dual-energy X-ray absorptiometry, Bone mineral density, Children

## Abstract

**Background:**

Patients with spinal muscular atrophy (SMA) are at risk of decreased bone mineral density (BMD). The bone health status of Chinese patients with SMA has been poorly studied. We aimed to evaluate the BMD of children with SMA types 2 and 3 in mainland China and investigate its influencing factors.

**Methods:**

Forty patients with a mean age of 5.5 years affected by SMA types 2 and 3 (*n* = 22 and *n* = 18, respectively) were enrolled between September 2017 and May 2019. Total body less head (TBLH) BMD, lumbar spine (LS) BMD, and body composition were measured using dual-energy X-ray absorptiometry (DXA). Serum bone metabolism markers and complete spinal radiographs were assessed. We utilized a linear regression model to explore the correlations between BMD and its related factors.

**Results:**

A total of 67.5% (27/40) of patients were diagnosed with low BMD and 2.5% (1/40) were diagnosed with osteoporosis. The TBLH BMD and LS BMD Z-scores in children with SMA type 2 were significantly lower than those with SMA type 3. Both TBLH and LS BMD Z-scores tended to increase with the change of SMA subtypes from 2a-3b. Vitamin D insufficiency and deficiency were found in 37.5% (15/40) of the patients. Serum Ca, phosphorus (P), alkaline phosphatase (ALP) and parathormone (PTH) levels were normal. There were no significant differences among the four subtypes in terms of all the serum bone metabolism markers. Phenotype was significantly associated with TBLH BMD and LS BMD Z-scores, and serum PTH levels were significantly associated with TBLH BMD Z-scores.

**Conclusions:**

Low BMD and osteoporosis were highly prevalent in mainland Chinese children with SMA types 2 and 3. Phenotype and serum PTH level might be the influencing factors of BMD. Regular monitoring of BMD by DXA scan and taking active interventions aim to SMA children with different types are important.

## Background

Spinal muscular atrophy (SMA) is a rare neurodegenerative disease characterized by the degeneration of the anterior horn of the spinal cord and medullary motor neurons, leading to progressive, symmetrical muscle weakness and muscle atrophy of the proximal limbs and trunk. The incidence of all types of SMA combined is around 1 in 12,000 live births [1]. The pathogenic mutation of survival motor neuron gene 1 (*SMN1*), located on chromosome 5q, results in insufficient expression of the survival motor neuron (SMN) protein [2]. *SMN2* is a paralogous gene that also encodes SMN protein, but only produces 10% of the level of functional full-length SMN protein [3]. The SMA phenotype is classified into four types (1 to 4) based on the age of onset and the maximum motor function achieved [4]. Infants with SMA type 1 are most severely affected. Median survival in “classic” SMA type 1 is 6 to 8 months [5]. SMA type 2 patients can sit upright but cannot stand or walk independently. SMA type 3 is a milder childhood-onset form, where patients can walk independently. SMA type 4 is an adult-onset disease with mild muscle weakness. According to the criteria for subclassification, we can also distinguish SMA subtypes 1a–1c, 2a and 2b, 3a and 3b [6, 7].

Muscular atrophy and chronic immobility may lead to low bone mineral density (BMD), osteoporosis, and an increased risk of fractures [8]. Therefore, skeletal system abnormalities are some of the most significant complications, and key factors in limiting the quality of life of children affected by SMA. The evaluation of bone health status has become an important part of SMA management. Published literatures have reported bone density data, fracture incidence, and treatment in children with SMA in different regions [9–11]. However, the BMD value is not known in mainland Chinese children with SMA. We evaluated bone density in Chinese children with SMA based on dual-energy X-ray absorptiometry (DXA) scanning in 40 patients with SMA types 2 and 3, obtained baseline data on BMD in different SMA subtypes, and analyzed its influencing factors in this population. This could lead to improved bone health management.

## Methods

### Study subjects

We enrolled children who were genetically confirmed to have 5q SMA with a homozygous deletion of exon 7 or 8, or both, at the Department of Neurology in Children’s Hospital Capital Institute of Pediatrics between September 2017 and May 2019. The inclusion criteria were: (1) no history of spinal trauma; (2) ability to complete the DXA measurement in the required horizontal position; and (3) no gene therapy. The exclusion criteria were: (1) treatment with drugs that affect bone metabolism (e.g. valproic acid, glucocorticoids, bisphosphonates); and (2) concomitant chronic disease that could affect bone metabolism (e.g. inflammatory bowel disease, pituitary disorders). The study was approved by the Ethics Committee of the Capital Institute of Pediatrics (No. SHERLL2017007). Written informed consent was obtained from the parents or guardians of the participating children. All study methods were carried out in accordance with relevant guidelines and regulations.

### Study design

#### Collection of clinical data

The attending physicians of the research team conducted detailed medical history consultations for the participants. This included the assessment of the symptom onset time, the maximum motor function that could be achieved, number and location of previous fractures (confirmed on bone radiographs and clinical records), medications, use of vitamin D and calcium (Ca) in the past 3 months, rehabilitation, and family history.

#### Anthropometry

Weight was measured to the nearest 0.1 kg in lightweight clothing without shoes on a calibrated digital scale. Standing height was measured with a stadiometer. A measuring board was used for patients unable to stand, as follows: the child was helped by a technician to lie supine, with the legs straight and well-aligned with the body, and the ankles as close together as possible. The footboard-headboard distance was accurately measured. For patients with scoliosis, we measured body length with a flexible ruler.

#### Genetic testing

Genomic DNA was extracted, and the multiplex ligation-dependent probe amplification technique (P060 kit, MRC, Amsterdam, The Netherlands) was used to detect the copy numbers of *SMN1* and *SMN2*.

#### DXA scanning

Whole-body scanning was performed using Hologic Discovery (A, W, and Wi) fan-beam densitometers (Hologic, Bedford, MA, USA). The coefficient of variation (CV) was used as a quality control procedure. The CVs of A, W, and Wi were 0.471, 0.302, and 0.358%, respectively. Measurement reports were prepared by a technician with DXA-training certification. All DXA values were analyzed using Hologic Apex version 4.0 following the manufacturer’s guidelines.

We measured values (based on the published reference standards of BMD and body composition for Chinese children aged 3–18 years [12, 13]) as follows:

1. BMD (g/cm^2^): Total body less head (TBLH) BMD and lumbar spine (LS) (L1–L4) BMD were measured according to the International Society for Clinical Densitometry (ISCD) recommendation. For most pediatric and adolescent patients, the posterior-anterior spine and TBLH are the preferred skeletal sites for measuring BMD using DXA [14]. TBLH BMD Z-scores (the number of standard deviations that a patient’s BMD differs from the average of a healthy control population of the same age and sex) were calculated under the 2017 “Bone mineral density reference standards for Chinese children aged 3–18” [12]. LS BMD Z-scores were determined with DXA using standards for American children [15] because there are no Chinese standards. Height Z-scores were calculated according to the growth standard value of Chinese children [16]. If a patient’s height Z-score was < − 1, the BMD Z-score was also corrected for the height Z-score, according to the ISCD indication for the measurement of pediatric BMD [17].
2. Body composition: Appendicular skeletal muscle mass (ASM), total mass (TM), and ASM weight ratio (ASMR) were calculated using the formula ASMR = ASM/TM. ASMR Z-scores were calculated according to a healthy control population of the same ethnicity, sex, and age [13].

#### Criteria for BMD determination

Decreased BMD in children can be classified as osteoporosis or low BMD according to the standards established by the ISCD in 2019 [18]. Diagnostic criteria for osteoporosis were: (1) the finding of one or more vertebral compression (crush) fractures in the absence of local disease or high-energy trauma; or (2) BMD Z-score ≤ − 2.0, and two or more long bone fractures by 10 years of age, or three or more long bone fractures up to 19 years of age. The diagnostic criterion for low BMD was a BMD Z-score ≤ − 2.0.

#### Laboratory analyses

Fasting blood collection was used to measure serum bone metabolism markers, including blood Ca, phosphorus (P), alkaline phosphatase (ALP), parathormone (PTH), and 25-OH-D (vitamin D) concentrations. In accordance with the American Academy of Pediatrics [19], serum 25-OH-D concentrations were defined as follows: sufficiency (> 50.0 nmol/L), insufficiency (37.5–50.0 nmol/L), deficiency (≤ 37.5 nmol/L), and severe deficiency (≤ 12.5 nmol/L) in the pediatric population.

#### Radiographic examination

Frontal and lateral images of the entire spinal column were obtained to evaluate compression fractures of the spine. Radiographs were evaluated by two pediatric radiologists.

### Statistical methods

The mean ± standard deviation values were used to describe quantitative data with a normal or symmetrical distribution, and the median (P25, P75) was used to describe data with a non-normal distribution. Independent-samples t-test or mann-whitney U test was used for comparisons between two groups. Proportions or percentages were used to describe qualitative data, and a chi-square was used for comparisons between two groups. Jonckheere-Terpstra test was used to assess the trend of SMA subtype 2a-3b with BMD and serum bone metabolism markers respectively. Statistical significance was defined as *p* < 0.05, two-sided.

A multiple linear regression model was built to explore influencing factors of BMD Z-score including sex, age at DXA scanning, disease course, phenotype, serum bone metabolic markers (PTH, 25-OH-D), and ASMR Z-score. The TBLH BMD and LS BMD Z-scores were included in the model separately. A collinearity diagnosis was carried out during the process of regression with a tolerance higher than 0.1. *P* < 0.05 being considered significant. Data were processed with SPSS (version 23.0, IBM Corp., Armonk, NY, USA).

## Results

### Demographic data and clinical characteristics

A total of 51 children with SMA were evaluated at our institution between September 2017 and May 2019; 40 patients met the inclusion criteria (male: *n* = 19; female: *n* = 21) and 11 patients were excluded (five were unable to complete the DXA measurement in the required horizontal position, three refused to undergo blood tests, and three dropped out). The demographics and clinical characteristics of the sample, by SMA type, are shown in Table 1. In all, 22 SMA type 2 (2a =11, 2b = 11) and 18 SMA type 3 (3a = 13, 3b = 5). There were no significant differences between SMA types 2 and 3 (*p* > 0.05) for sex distribution, age at DXA scanning, and disease course. No patient had used vitamin D and Ca supplements regularly in the previous 3 months. Only 30% (12/40) of patients had undergone formal rehabilitation training.

**Table 1:** Demographics and BMD data of patients with SMA

	SMA 2 (*n* = 22)	SMA 3 (*n* = 18)	t/z/χ^2^	*p*-Value
Sex (male/female)	8/14	11/7	2.431	0.119
Age (y) at DXA	5.5 (4.1, 9.5)	5.4 (4.3, 3.9)	− 0.122	0.904
Disease course (y)	4.5 (3.2, 8.9)	3.9 (2.1, 5.4)	−1.251	0.219
TBLH BMD Z-scores	−3.7 ± 1.6	−2.0 ± 1.7	3.344	0.002
LS BMD Z-scores	−1.9 ± 1.2	−0.6 ± 1.4	3.266	0.002
ASMR Z-scores	−3.6 ± 1.1	−2.7 ± 1.3	2.476	0.018
Low BMD (%)	86.4	44.4	6.955	0.008
Osteoporosis(n)	0	1		

Data are shown as mean ± standard deviation or median (interquartile range). *BMD* bone mineral density, *SMA* spinal muscular atrophy, *DXA* dual-energy X-ray absorptiometry, *TBLH* total body less head, *LS* lumbar spine, *ASMR* appendicular skeletal muscle mass weight ratio

### BMD densitometry

**Fig. 1:**
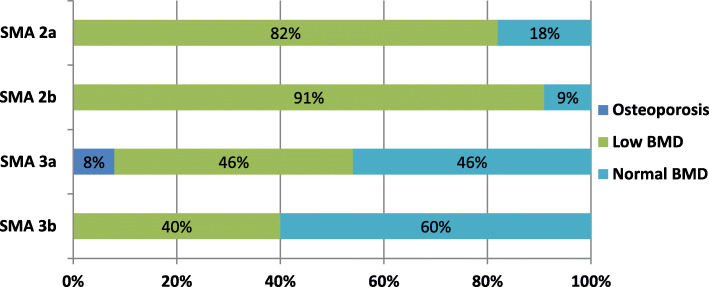
Distribution of low BMD and osteoporosis in different SMA subtypes. For patients with SMA 2a, 82% (9/11) were diagnosed with low BMD, 18% (2/11) were normal. For SMA 2b, 91% (10/11) were diagnosed with low BMD, 9% (1/11) were normal. For SMA 3a, 46% (6/13) were diagnosed with low BMD, 8% (1/13) were diagnosed with osteoporosis, 46% (6/13) were normal. For SMA 3b, 40% (2/5) were diagnosed with low BMD, 60% (3/5) were normal. BMD: bone mineral density; SMA: spinal muscular atrophy

DXA data were available for all study patients. The mean TBLH BMD Z-score of the 40 patients was − 3.0 ± 1.8, and the mean LS BMD Z-score was − 1.3 ± 1.4. Children with SMA type 2 had significantly lower TBLH BMD and LS BMD Z-scores than those with SMA type 3 (*P* = 0.002, *P* = 0.002, respectively). In 18 patients with SMA type 3, 12 carried three *SMN2* copies and 6 carried four copies. There were no significant differences between these two groups in terms of TBLH BMD and LS BMD Z-scores (*P* = 0.375, *P* = 1.000, respectively). According to the 2019 ISCD standard, 67.5% (27/40) of the patients were diagnosed with low BMD, 2.5% (1/40) were diagnosed with osteoporosis, and only 30% (12/40) had BMD in the normal range. For SMA type 2 patients, 86.4% (19/22) of them were diagnosed with low BMD. For SMA type 3 patients, 44.4% (8/18) were diagnosed with low BMD. There was significant difference between the two groups (*p* = 0.008) (Table 1). Distribution of BMD status in different SMA subtypes was show in Fig. 1. Jonckheere-Terpstra test demonstrated that there was significant trend of SMA subtypes with TBLH BMD Z-scores and LS BMD Z-scores (*p* = 0.001, *p* < 0.001 respectively) (Table 2). Both TBLH and LS BMD Z-scores tended to increase with the change of SMA subtypes from 2a-3b (Fig. 2).

**Table 2:** Comparison of BMD among different SMA subtype

	SMA 2a (*n* = 11)	SMA 2b (*n* = 11)	SMA 3a (*n* = 13)	SMA 3b (*n* = 5)	*p*-value^#^
TBLH BMD Z-scores	−4.1 ± 1.4	−3.5 ± 1.8	−2.3 ± 1.7	−1.3 ± 1.6	0.001
LS BMD Z-scores	−2.4 ± 1.2	−1.7 ± 1.3	− 0.6 ± 1.2	−0.1 ± 0.6	< 0.001

Data are shown as mean ± standard deviation. *BMD* bone mineral density, *SMA* spinal muscular atrophy, *TBLH* total body less head, *LS* lumbar spine

# *p*-values were calculated by Jonckheere-Terpstra test

**Fig. 2:**
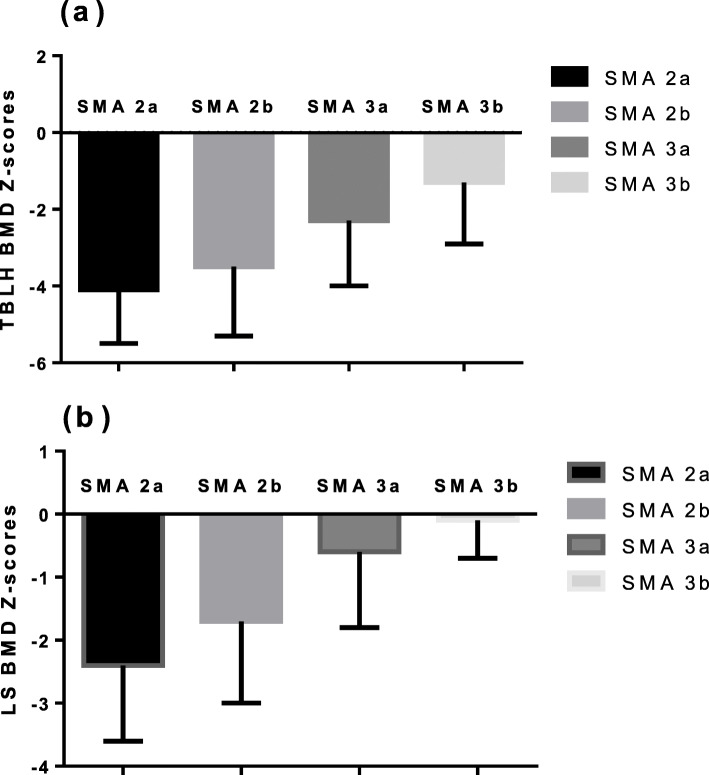
BMD Z-scores trend of change in different SMA subtypes. Data are shown as mean ± standard deviation. With the change of SMA subtype from 2a -3b, the mean values of both TBLH BMD and LS BMD were gradually increasing. BMD: bone mineral density; SMA: spinal muscular atrophy; TBLH: total body less head; LS: lumbar spine

### Fractures

None of the 40 children had vertebral fractures. This was confirmed by spinal radiographs. Two patients (5.0%) had histories of long bone fractures, including one child of SMA 3a who had two low-trauma fractures of the proximal humerus during activity at the age of 2 years, and one child of SMA 2b who had a right femur fracture at the age of 8 years.

### Body composition

DXA body composition measurements were performed in all 40 patients, and the results are shown in Table 1. Our results showed that the mean ASMR Z-score was − 3.2 ± 1.2, which was lower than that of healthy children (ASMR Z-score from − 2.0 to 2.0). Patients with SMA type 2 had significantly lower ASMR Z-scores than those with SMA type 3 (*P* = 0.018).

### Laboratory test results

The mean levels of serum Ca, P, ALP, PTH and 25-OH-D in different SMA subtypes are shown in Table 3. The ALP levels were in the normal range in the 40 patients, and serum Ca was slightly below the normal range in only one child (3%). Serum phosphate was slightly above the normal range in five children (13%), and serum PTH was slightly below the normal range in only one child (3%). The mean level of serum 25-OH-D was 53.93 ± 19.68 nmol/L. Among the 40 patients, the levels of serum 25-OH-D fulfilled the criteria for insufficiency (range, 37.5–50.0 nmol/L) in six children (15%), deficiency (≤ 37.5 nmol/L) in nine children (22.5%), and there was no severe deficiency. In 25 (62.5%) cases, the vitamin D values were within the normal range. Jonckheere-Terpstra test demonstrated that there were no significant differences among the four subtypes in terms of all the serum bone metabolism markers. (*P* > 0.05) (Table 3).

**Table 3:** Comparison of serum bone metabolism markers between SMA subtypes

	SMA 2a (*n* = 11)	SMA 2b (*n* = 11)	SMA 3a (*n* = 13)	SMA 3b (*n* = 5)	*p*-value^#^
Ca (mmol/L)	2.42 ± 0.07	2.47 ± 0.11	2.40 ± 0.11	2.40 ± 0.01	0.465
P (mmol/L)	1.80 ± 0.18	1.76 ± 0.17	1.79 ± 0.20	1.76 ± 0.21	0.884
ALP(U/L)	175.91 ± 52.42	151.55 ± 32.35	167.69 ± 32.71	174.40 ± 34.21	0.951
PTH (pg/ml)	23.13 ± 6.47	30.29 ± 13.59	27.95 ± 12.83	32.46 ± 7.94	0.147
25-OH-D (nmol/L)	56.77 ± 14.52	47.97 ± 14.62	59.43 ± 26.51	46.49 ± 17.81	0.559

Data are shown as mean ± standard deviation. *SMA* spinal muscular atrophy, *ALP* alkaline phosphatase, *PTH* parathormone

# *p*-values were calculated by Jonckheere-Terpstra test

### Influencing factors of BMD

Sex, age at DXA scanning, disease course, phenotype, serum bone metabolism markers (PTH, 25-OH-D), ASMR Z-scores, and BMD Z-scores were evaluated with a linear regression model. The results showed that phenotype and PTH levels were significantly associated with the TBLH BMD Z-scores in patients with SMA (*P* = 0.008, *P* = 0.015, respectively) (Table 4); and phenotype was significantly associated with the LS BMD Z-scores (*P* = 0.009) (Table 5).

**Table 4:** Influencing factors for TBLH BMD Z-scores in children with SMA

	Coefficients	Standardized Coefficients	t	*p*-value
Sex	−0.574	−0.159	−0.943	0.353
Age at DXA	0.008	0.014	0.058	0.954
Disease course	−0.149	−0.238	−0.946	0.351
Phenotype	1.599	0.441	2.847	0.008
PTH	0.068	0.417	2.572	0.015
25-OH-D	0.004	0.040	0.214	0.832
ASMR Z-score	−0.015	−0.010	−0.057	0.955

*R*^*2*^ = 0.433

*TBLH* total body less head, *BMD* bone mineral density, *SMA* spinal muscular atrophy, *DXA* dual-energy X-ray absorptiometry, *PTH* parathormone, *ASMR* appendicular skeletal muscle mass weight ratio

**Table 5:** Influencing factors for LS BMD Z-scores in children with SMA

	Coefficients	Standardized Coefficients	t	*p*-Value
Sex	−0.591	−0.210	−1.143	0.262
Age at DXA	0.054	0.130	0.485	0.631
Disease course	0.016	0.033	0.120	0.905
Phenotype	1.320	0.466	2.762	0.009
PTH	0.041	0.326	1.848	0.074
25-OH-D	0.018	0.249	1.222	0.231
ASMR Z-scores	0.026	0.022	0.117	0.908

*R*^*2*^ = 0.327

*LS* lumbar spine, *BMD* bone mineral density, *SMA* spinal muscular atrophy, *DXA* dual-energy X-ray absorptiometry, *PTH* parathormone, *ASMR* appendicular skeletal muscle mass weight ratio

## Discussion

In this study, we found that low BMD and osteoporosis were prevalent in children with SMA types 2 and 3, and significant differences in BMD were observed in different types. More than one-third of the patients were diagnosed with Vitamin D insufficiency or deficiency, but serum Ca, P, ALP, and PTH were at approximately normal levels. The results of our statistical model showed that phenotype and serum PTH level are significantly correlated with BMD.

Patients with SMA have an increased probability of pathologically low bone mineral content due to muscle atrophy, limited development of gross motor functions, long-term low activity, insufficient vitamin D intake. This increases the risk of fragility fractures, severely affecting the quality of life. Skeletal system complications have been reported in children with SMA types 1–3. In 1986, Burke et al. [20] first presented three cases of children with SMA type 1 with multiple perinatal fractures. In 2012, Poruk et al. [21] reported that the mean values of whole-body BMD and LS BMD of 47 patients with SMA type 1 were both much lower than those of age-matched healthy controls. An increasing number of BMD studies have included children with SMA types 2 and 3 in recent years. Vai et al. [22] confirmed that the LS bone mineral apparent density Z-scores significantly decreased below − 1.5 in 50% of children with SMA types 2 and 3, and fractures occurred in 36.7% of patients, including four patients with peripheral fractures and seven with vertebral fractures. Wasserman et al. [23] reported a BMD Z-score below − 2.0 in 85% of 62 patients with SMA types 1–3, and osteoporosis was diagnosed in 12.9% of these patients. Furthermore, fractures and osteoporosis could occur even in younger patients aged 3–4 years. In our cohort, 70% of the patients had BMD Z-scores ≤ − 2.0, consistent with the results of these previous studies. It indicates that low BMD is also a frequent occurrence in mainland Chinese patients. So, regular DXA scan in children with SMA types 2 and 3 is necessary. In the latest International Management Consensus [24], annual DXA to monitor bone density in patients with SMA (types 2 and 3) is recommended. If the patient develops osteoporosis and receives treatment that affects the skeleton, BMD can be reassessed after 6 months, as a minimum interval [18].

Previous studies reported the factors associated with BMD in children with SMA, including age, type, course of disease, motor function, serum vitamin D concentrations and so on [10, 22, 23]. Though the sample size for our study was not large, we explored the potential influencing factors of BMD by statistical models in the further research of such a rare disease. The result indicated that phenotype was assumed to be an influencing factor of the TBLH BMD and LS BMD Z-scores. Compared between SMA type 2 and SMA type 3, both the TBLH BMD and LS BMD Z-scores of children with SMA type 2 were significantly lower than those of type 3. In patients diagnosed with low BMD, 2a and 2b were the primary subtypes. Our analysis demonstrated a pronounced trend of BMD reduction with phenotypes severity. In other words, the maximum motor function achieved can affect the patient’s BMD. Wasserman et al. [23] also reported that patients with SMA type 1 had significantly lower BMD Z-scores at all skeletal sites compared to those with SMA type 2 or 3. This study showed that the more severe the phenotype, the lower the BMD Z-score. The effect of the severity of the phenotype on BMD may be related to many factors. Low BMD is related to the degree of muscle atrophy and motor function. The more severe the phenotype, the more obvious the muscle atrophy and the lower the motor function scores. Behringer et al. [25] revealed that ASM atrophy in SMA type 2 was more obvious than that in SMA type 3, while weight-bearing activities and muscle traction could directly affect the increase of BMD. In addition, the survival motor neuron (SMN) protein may directly influence BMD. Khatri et al. [26] revealed that the reduction in BMD in pediatric patients with SMA tends to be more pronounced than that in patients with other neuromuscular diseases. A previous study confirmed that the SMN protein plays an important role in bone remodeling and affects bone metabolism by regulating the expression of osteoclast stimulating factor by osteoclasts [27]. In patients with SMA, the more severe the phenotype, the lower the amount of SMN protein. Therefore, a high incidence of low BMD and fractures in patients with SMA may not simply be attributed to muscle weakness and lack of exercise but is one of the primary symptoms of the disease itself [28, 29]. Thus, we believe that more attention should be paid to the bone health status of children with SMA type 2 in clinical practice and suggest closer monitoring of BMD in this group than in SMA type 3 patients. Maintaining maximum motor function through aggressive treatment and rehabilitation is critical.

Children with SMA have a high incidence of fragility fractures. But only 5% (2/40) of children with SMA in our study had fractures, which was significantly lower than the fracture rate of 36–46% reported by other studies [22, 30, 31]. The low rate of fractures in our study patients may be related to their decreased participation in outdoor activities and rehabilitation. In our cohort, the patients with SMA were over-protected by their parents and seldom went outdoors to avoid possible injury, and only 30% (12/40) of the children had visited a formal rehabilitation department for regular physical treatment for 1–3 years. The fractures in our patients occurred in children with regular long-term rehabilitation training and activities. A previous study has reported that fractures in patients with SMA and osteoporosis may occur during the regular rehabilitation process [11]. With the development of multidisciplinary management and intrathecal administration of nusinersen in China, patients will inevitably face more rehabilitation training and a return to social life, and the proportion of fractures in Chinese patients may increase. Due to the high prevalence of low BMD in this population, we recommend the regular monitoring of BMD for Chinese patients with SMA in the future, and the appropriate exercise and rehabilitation methods must be arranged according to their BMD data.

A low 25-OH-D level was found in 37.5% of our patients with SMA types 2 and 3, consistent with the findings of the study in other country [10]. Our study also showed that the serum PTH level was correlated with TBLH BMD in the linear regression model. Since PTH can promote bone resorption, elevated PTH levels can lead to bone density reduction. It is known that there is a negative relationship between vitamin D and PTH levels. Hence, vitamin D deficiency must be corrected to avoid an abnormal increase in bone resorption due to increased PTH secretion. Notably, vitamin D is a steroid hormone that can promote the absorption of Ca and P by small intestinal mucosal cells, thereby increasing blood Ca and P concentrations, which are beneficial to new bone formation and calcification, thus playing an important biological role in bone health. The consensus recommendation among experts is that Vitamin D blood levels and intake should be monitored, at least, annually and supplements should be given in the presence of low levels or osteopenia [24]. However, based on our statistical model, there was no significant association between 25-OH-D level and BMD. So, our opinion is that reduced serum vitamin D concentrations is not an indication to increase the number of BMD assessments. Moreover, we also do not recommend routine serum Ca, P or ALP screening in SMA children with low bone density or osteoporosis, in order to limit the assessment burden.

Although our study was the first to report a comprehensive view of BMD and fracture history across pediatric SMA types 2 and 3 in mainland China, there were still several limitations. Firstly, children with SMA type 1 were not included in our study, this leaded to incomplete bone mineral density data. Secondly, the small sample size may cause data bias. Finally, our report cross-sectionally examined bone mineral density and lacked long-term monitoring and follow-up.

## Conclusion

In conclusion, low BMD and osteoporosis were highly prevalent in mainland Chinese children with SMA types 2 and 3. Phenotype and serum PTH level might be the most important influencing factors of BMD. Regular monitoring of BMD by DXA scan and taking active interventions aim to SMA children with different types are necessary in Chinese children with SMA types 2 and 3. For better multidisciplinary management and individual treatment, a future expanded sample size cohort study of BMD is warranted for Chinese patients with SMA.

## Data Availability

The datasets used and analyzed during the current study are available from the corresponding author on reasonable request.

## Abbreviations

SMA: Spinal muscular atrophy
TBLH: Total body less head
LS: Lumbar spine
BMD: Bone mineral density
DXA: Dual-energy X-ray absorptiometry
SD: Standard deviation
MLPA: Multiplex ligation-dependent probe amplification
SMN: Survival motor neuron
PTH: Serum parathormone
ASM: Appendicular skeletal muscle mass
TM: Total mass
ASMR: ASM ratios
ISCD: International Society for Clinical Densitometry
